# Improved time-to-detection with the new formulation of BD BACTEC Plus Aerobic/F Culture Vials: a real-world evidence study

**DOI:** 10.1128/spectrum.01969-25

**Published:** 2025-09-12

**Authors:** Kayla Van Benten, Mondraya Howard, Valentin Parvu, Chris Massey, Stephanie Frey

**Affiliations:** 1 Becton, Dickinson and Company, Diagnostic Solutions 713731, Sparks, Maryland, USA; 2 Laboratory Department, Penn Medicine Lancaster General Health 209639 , Lancaster, Pennsylvania, USA; Johns Hopkins University, Baltimore, Maryland, USA

**Keywords:** bloodstream infection, blood culture vials, BD BACTEC media, automated blood culture, time-to-detection

## Abstract

BD BACTEC Plus Aerobic/F Culture Vials were modified to reduce time-to-detection (TTD) of bacteria and yeast in blood specimens. Analytical testing involved seeded blood specimens in either predicate plus (N = 500) and modified plus (N = 500) media. Clinical testing used positive aerobic vial results from BD EpiCenter System Software for two periods: predicate plus (N = 1,075) and modified plus medium (N = 873). TTD was measured from the start of vial incubation to microorganism growth detection on BD BACTEC FX. Wilcoxon rank-sum and Chi-square (χ²) tests were used to compare TTD and 48-hour positivity. Modified plus reduced TTD by 2.6 hours (*P* < 0.001) in analytical testing and 2.9 hours (*P* < 0.05) in clinical testing. Significant TTD reductions were observed for *E. coli*, *E. faecalis*, *S. epidermidis*, and *P. mirabilis*. The incidence of late positives (detection after 48 hours) decreased from 10.5% to 6.9%. Modified plus media significantly reduces TTD and the number of late positives for a range of microorganisms.

**IMPORTANCE:**

Faster detection of infectious organisms can help improve patient prognosis. Recently, the formulation of aerobic culture vial media was modified for blood culture on the BD BACTEC instrument. The work presented here provides compelling evidence that the new aerobic media formulation improves time-to-detection for bloodstream infections. This has the potential to impact the healthcare treatment of millions of patients each year without requiring changes in clinical workflow. Faster time to detection can lead to faster organism identification and antimicrobial susceptibility testing (when appropriate). This report provides a comprehensive comparison of the novel media formulation with the predicate formulation for detecting multiple organism genera in both analytical and prospective clinical studies.

## INTRODUCTION

Bloodstream infection (BSI) is a potentially life-threatening condition that can severely impact patient health and outcomes (1). BSIs often lead to prolonged hospital stays, frequently result in intensive care unit admissions, cause severe complications such as sepsis and organ failure, and significantly increase the cost of patient care (2–10). Successful patient outcomes are jeopardized when the causative pathogens are not identified rapidly for appropriate therapy (11, 12). Automated blood culture platforms have a high analytical sensitivity, compared to traditional microbiology methods, and can significantly reduce the time-to-detection (TTD) of bacterial growth (13–15). In addition, automated systems based on technologies such as monitoring colorimetric or fluorescence changes due to microorganism CO_2_ production can facilitate recovery of a wider range of microorganisms (14). By reducing TTD, automated systems provide an important, cost-effective means of BSI diagnosis (16).

Whereas the most widely used medium for aerobic blood culture vials is soybean casein digest (SCD) broth (17), other media such as Brain Heart Infusion (BHI) and supplemented peptone broths are also used to recover fastidious microorganisms (18). To improve detection of slow-growing organisms in blood cultures, most commercial media also contain a proprietary combination of various nutrients and other growth factors (19). BD BACTEC Plus Aerobic/F Culture Vials (Becton, Dickinson and Company; BD Life Sciences—Diagnostic Solutions), used in conjunction with the BD BACTEC FX instrument (“BACTEC FX”; Becton, Dickinson and Company; BD Life Sciences—Diagnostic Solutions), facilitate a qualitative procedure for the aerobic culture and recovery of microorganisms (bacteria and yeast) from blood. In April 2023, a formulation modification to BD BACTEC Plus Aerobic/F Culture Vials (“BACTEC Plus Aerobic/F media”; Becton, Dickinson and Company; BD Life Sciences—Diagnostic Solutions) was released to improve TTD. The modified formulation includes a proprietary combination of inorganic compounds to enhance the growth and detection of target microorganisms. In this study, we evaluated the impact of the modified formulation of BACTEC Plus Aerobic/F media on blood culture TTD and positivity rate.

## MATERIALS AND METHODS

This report includes results from two separate experimental designs. The first includes analytical testing of bacterial and yeast organisms at known concentrations to directly compare TTD between BD BACTEC Plus Aerobic/F Culture Vials (“predicate plus media”; Becton, Dickinson and Company; BD Life Sciences—Diagnostic Solutions) and BD BACTEC Plus Aerobic/F Culture Vials containing a modified formulation (“modified plus media”; Becton, Dickinson and Company; BD Life Sciences—Diagnostic Solutions). The second component of this report involves collection and analyses of retrospective data in combination with prospectively collected data, from a single site (Lancaster General Health, Lancaster, PA), in order to compare TTD before (predicate plus media) and after the reformulation (modified plus media). De-identified samples from patients who had undergone routine blood culture collection at the study site were used. Pediatric patients were excluded from the study. This study was conducted according to principles outlined by the Declaration of Helsinki and Good Clinical Practice. Institutional Review Board (IRB) clearance of the data utilized in this study was received from Advarra (Pro0081240), and the manuscript was written following GPP3 guidelines.

The BD BACTEC FX instrument (“BACTEC”; Becton, Dickinson and Company; BD Life Sciences—Diagnostic Solutions) is designed for the rapid detection of bacteria and fungi in clinical specimens, blood, and blood products. Specimens are obtained from patients or bagged blood/blood products and injected directly into BD BACTEC Plus Aerobic/F Culture Vials, which are then placed in the instrument for incubation and testing. Each vial contains a chemical sensor that detects increases in CO_2_ produced by the growth of microorganisms. The instrument monitors the sensor every 10 minutes for an increase in its fluorescence, which is proportional to the amount of CO_2_ present. If microorganisms are present in the test sample, CO_2_ is produced as substrates are metabolized in the vial. Analysis of the rate and amount of CO_2_ increase enables the BACTEC instrument to determine whether a vial is positive, i.e., the test sample contains viable organisms. Resins have been incorporated into plus media to enhance the recovery of organisms without the need for special processing.

### Analytical study

Paired analytical specimens (10 mL bagged blood specimens seeded with isolates of known concentration) were prepared for incubation in either predicate or modified plus media (N = 500 for each media type). Bagged blood for analytical experiments was obtained from BioIVT (Westbury, NY). Frozen bacterial or yeast vial stocks, consisting of a combination of clinical strains and well-characterized control strains, were thawed at room temperature, streaked onto one to three agar plates (consisting of organism-appropriate medium) with a sterile 10 µL inoculating loop (“first subculture”). The plate was then incubated at 35 ± 2°C and monitored until visible growth of individual colonies was observed. Incubation time varied by organism or strain but was typically 24–48 hours. Colonies were then subcultured onto fresh agar plates (one to three colonies) with a 10 µL inoculating loop, and the plates were incubated as with the initial subculture (“second subculture”). All colonies (bacterial and yeast) were harvested using a sterile cotton-tipped applicator and then suspended in saline to a turbidity of 1.0 McFarland (except *H. influenzae* at 0.5 McFarland and *S. pneumoniae* at 1.3 McFarland), which corresponds to ~3 × 10^8^ CFU/mL for bacteria. For yeast, 0.5 McFarland corresponds to ~1 × 10^7^ CFU/mL for *C. glabrata* and 5 × 10^6^ CFU/mL for other yeast. Suspensions were then serially diluted in saline to a final concentration of 30 CFU/mL for bacteria and 50 CFU/mL for yeast species. A 0.1 mL aliquot was added, using 1-cc syringes with 25–28-gauge needles, to BACTEC vials with a blood volume of either three or 10 mL. For all specimens, the final inoculum was verified by quantitation of colony-forming units, following the spread of a known volume of the inoculum suspension onto appropriate plated media with subsequent incubation.

### Clinical study

Blood collection, vial inoculation, and incubation were performed according to BACTEC FX instructions for use and the site’s standard of care. Positive aerobic vial results were extracted from the site’s EpiCenter system for two separate periods: using predicate plus media (phase 1; from January 1, 2023, to June 30, 2023) and modified plus media (phase 2; from October 20, 2023, to March 20, 2024). Phase 1 included at least six months of data immediately prior to conversion, while phase 2 included data obtained at least six months post-conversion. TTD was measured as the interval between the start of vial incubation to detection of microorganism growth. Organism identification during clinical specimen workflow was accomplished using matrix-assisted laser desorption ionization–time of flight mass spectrometry. Data on fill volume were obtained from EpiCenter, and positivity and contamination rates were obtained from the study site’s LIS.

### Statistical analysis

Data were analyzed using Excel, Minitab, and Statistica packages. Paired specimen data for predicate and modified plus media groups were used for analytical testing. For analysis from the clinical study, the Mann–Whitney *U* test for nonparametric data was used to compare differences between independent groups, assuming a non-normal sample distribution. For all statistical analyses, the null hypothesis was that the TTDs associated with predicate plus media and modified plus media groups were equal. The threshold for significance was a *P*-value < 0.05. The minimum group number to be included in any analysis was *n* = 15.

## RESULTS

**Fig 1:**
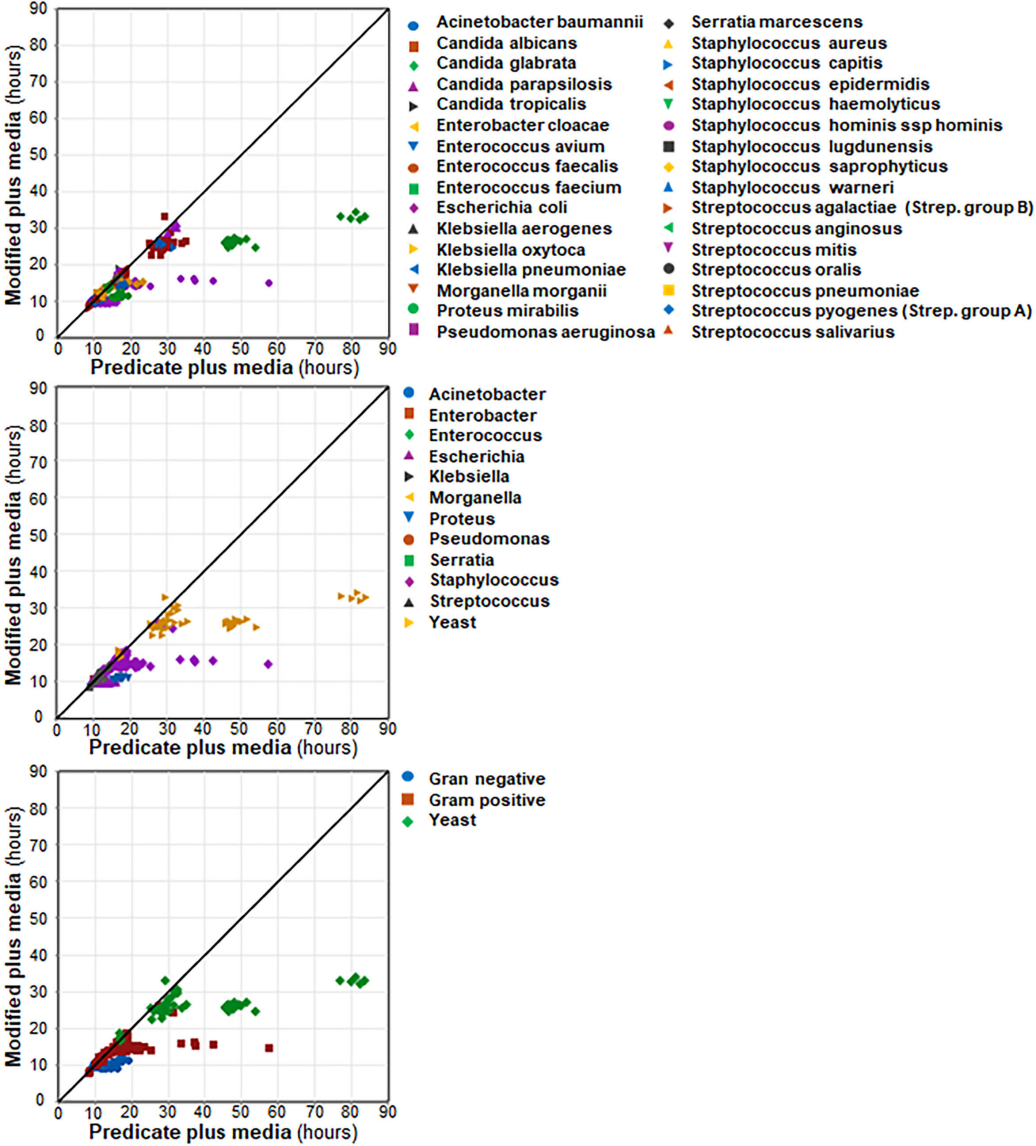
Scatter plots for TTD associated with paired predicate plus and modified plus media specimens during analytical testing. Analyses are shown at species level, genera, and grouped by Gram stain (or yeast).

For all organisms combined, a statistically significant difference was observed in the mean TTD between the predicate (16.2 hours) media and the modified plus (13.6 hours) media (mean difference of 2.5 hours; *P* < 0.001; Table 1). The scatterplot in Fig. 1 demonstrates the shift in TTD for modified plus media at the species and genus level and by Gram stain (or yeast) classification. For yeasts as a group, modified plus media had a mean difference of −12.9 hours in TTD when compared with predicate plus media (*P*-value < 0.001) (Table 1). Modified plus media showed significantly lower TTD compared to predicate plus media for detection of *C. albicans*, *C. glabrata*, and *C. parapsilosis* (Table S1). In addition, significant differences in mean TTD were observed with modified plus media compared to predicate plus media in both Gram-positive (−1.6 hours) and Gram-negative (−1.2 hours) organisms (Table 1; Fig. 1). Lower TTD was observed with modified plus media across several genera, including *Enterococcus* (mean difference of −0.9 hours; *P* < 0.001), *Staphylococcus* (mean difference of −2.0 hours; *P* < 0.001), *Escherichia* (mean difference of −1.4 hours; *P* < 0.001), *Klebsiella* (mean difference of −0.6 hours; *P* < 0.001), *Morganella* (mean difference of −0.7 hours; *P* < 0.01), and *Proteus* (mean difference of −5.5 hours; *P* < 0.01) (Table 1). For 20 of the 32 individual organisms tested, modified plus media resulted in a significantly lower TTD compared to predicate plus media; conversely, none of the individual organisms tested were associated with a significantly lower TTD for predicate plus media compared to modified plus media (Table S1). Incubation of 3 mL seeded blood specimens yielded results similar to those obtained with a 10 mL blood volume (Table S2 and Table S3).

**TABLE 1:** Comparison of predicate and modified plus media in analytical testing; time-to-detection (hours) results by genera, yeast, and Gram stain category (blood volume tested = 10 mL)

Category	Media type	*N*	Mean (SD)	Median (25%, 75%)	*P*-value
Overall	Predicate plus	500	16.2 (10.4)	12.2 (11.0, 17.2)	<0.001
	Modified plus	500	13.6 (5.1)	11.8 (10.1, 15.3)	
*Acinetobacter*	Predicate plus	5	9.3 (0.1)	9.3 (9.2, 9.3)	0.181
	Modified plus	5	9.3 (0.0)	9.3 (9.3, 9.3)	
*Enterobacter*	Predicate plus	5	10.2 (0.2)	10.1 (10.1, 10.3)	0.576
	Modified plus	5	10.3 (0.2)	10.1 (10.1, 10.5)	
*Enterococcus*	Predicate plus	30	13.2 (2.8)	11.5 (11.1, 16.7)	<0.001
	Modified plus	30	12.3 (1.6)	11.4 (11.1, 13.8)	
*Escherichia*	Predicate plus	95	11.0 (1.8)	10.4 (9.6, 11.8)	<0.001
	Modified plus	95	9.6 (0.3)	9.6 (9.3, 9.9)	
*Klebsiella*	Predicate plus	40	11.0 (0.9)	10.9 (10.3, 11.7)	<0.001
	Modified plus	40	10.4 (0.6)	10.5 (10.0, 10.9)	
*Morganella*	Predicate plus	5	11.9 (0.4)	11.9 (11.6, 12.2)	<0.01
	Modified plus	5	11.2 (0.1)	11.2 (11.1, 11.2)	
*Proteus*	Predicate plus	10	16.8 (1.3)	16.8 (15.6, 17.7)	<0.0001
	Modified plus	10	11.3 (0.4)	11.2 (10.9, 11.7)	
*Pseudomonas*	Predicate plus	15	16.9 (0.5)	16.7 (16.4, 17.3)	0.203
	Modified plus	15	17.1 (0.6)	17.1 (16.4, 17.6)	
*Serratia*	Predicate plus	5	12.2 (0.1)	12.3 (12.1, 12.3)	0.190
	Modified plus	5	12.4 (0.2)	12.5 (12.2, 12.5)	
*Staphylococcus*	Predicate plus	190	16.2 (5.9)	15.9 (11.9, 18.1)	<0.001
	Modified plus	190	14.2 (2.7)	14.1 (11.9, 15.6)	
*Streptococcus*	Predicate plus	50	11.0 (1.5)	11.2 (10.2, 11.9)	0.493
	Modified plus	50	10.9 (1.7)	11.2 (10.0, 11.8)	
Yeast	Predicate plus	50	39.0 (17.5)	31.7 (28.1, 48.1)	<0.001
	Modified plus	50	26.1 (4.0)	26.1 (24.6, 27.3)	
Gram-negative	Predicate plus	180	11.8 (2.5)	10.9 (10.0, 12.6)	<0.001
	Modified plus	180	10.6 (2.1)	9.9 (9.4, 10.8)	
Gram-positive	Predicate plus	270	14.9 (5.5)	12.6 (11.5, 17.3)	<0.001
	Modified plus	270	13.3 (2.7)	12.5 (11.6, 15.1)	

**Fig 2:**
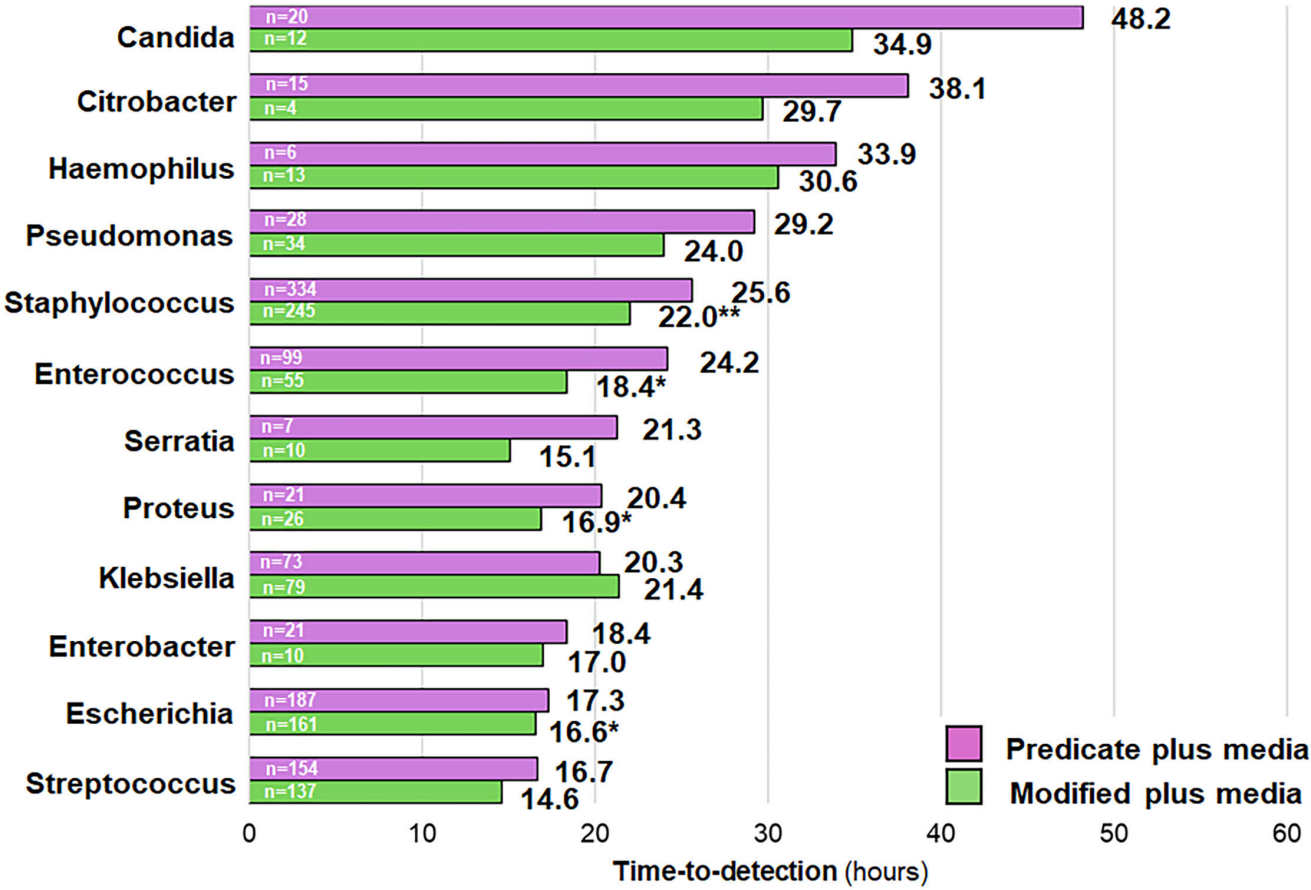
Comparison of TTD results either before or after the implementation of modified plus media. Positive predicate plus and modified plus media results were extracted from the EpiCenter microbiology data management system for time periods corresponding to incubation in either predicate plus (01/01/2023 to 06/30/2023) or modified plus media (10/20/2023 to 03/20/2024). Data are presented for mean TTD. Wilcoxon rank-sum nonparametric test was used to compare the two study periods. Abbreviations: TTD, time-to-detection. * indicates a *P*-value < 0.05. ** indicates a *P*-value < 0.01.

**Fig 3:**
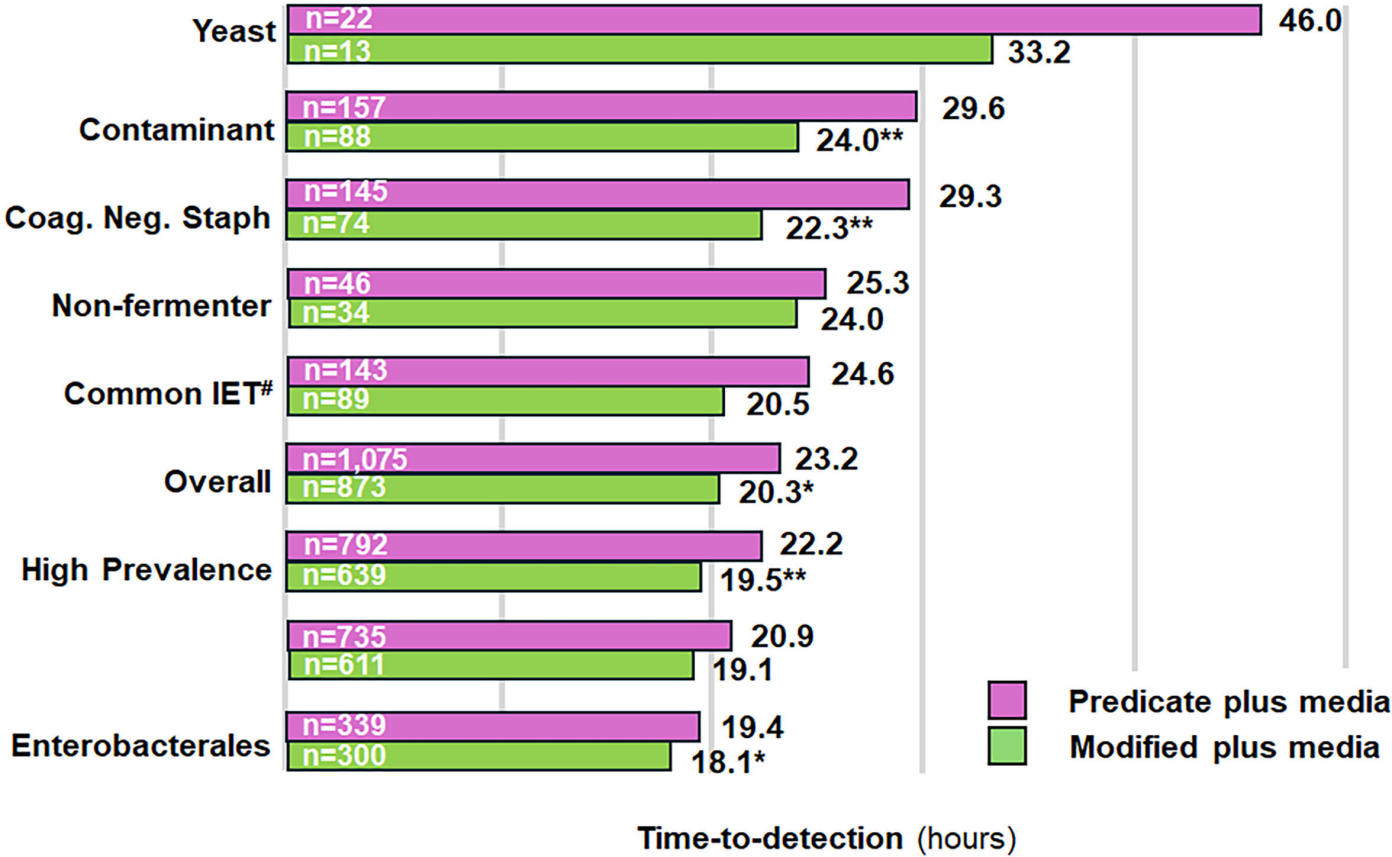
Comparison of TTD results either before or after the implementation of modified plus media. Positive results were extracted from the EpiCenter microbiology data management system for time periods corresponding to incubation in either predicate plus (01/01/2023 to 06/30/2023) or modified plus media (10/20/2023 to 03/20/2024). Data are presented for mean TTD. Wilcoxon rank-sum nonparametric test was used to compare the two study periods. Abbreviations: TTD, time-to-detection; IET, inappropriate empirical treatment. * indicates a *P*-value < 0.05. ** indicates a *P*-value < 0.01. #Kadri et al. (2021) (20).

**Fig 4:**
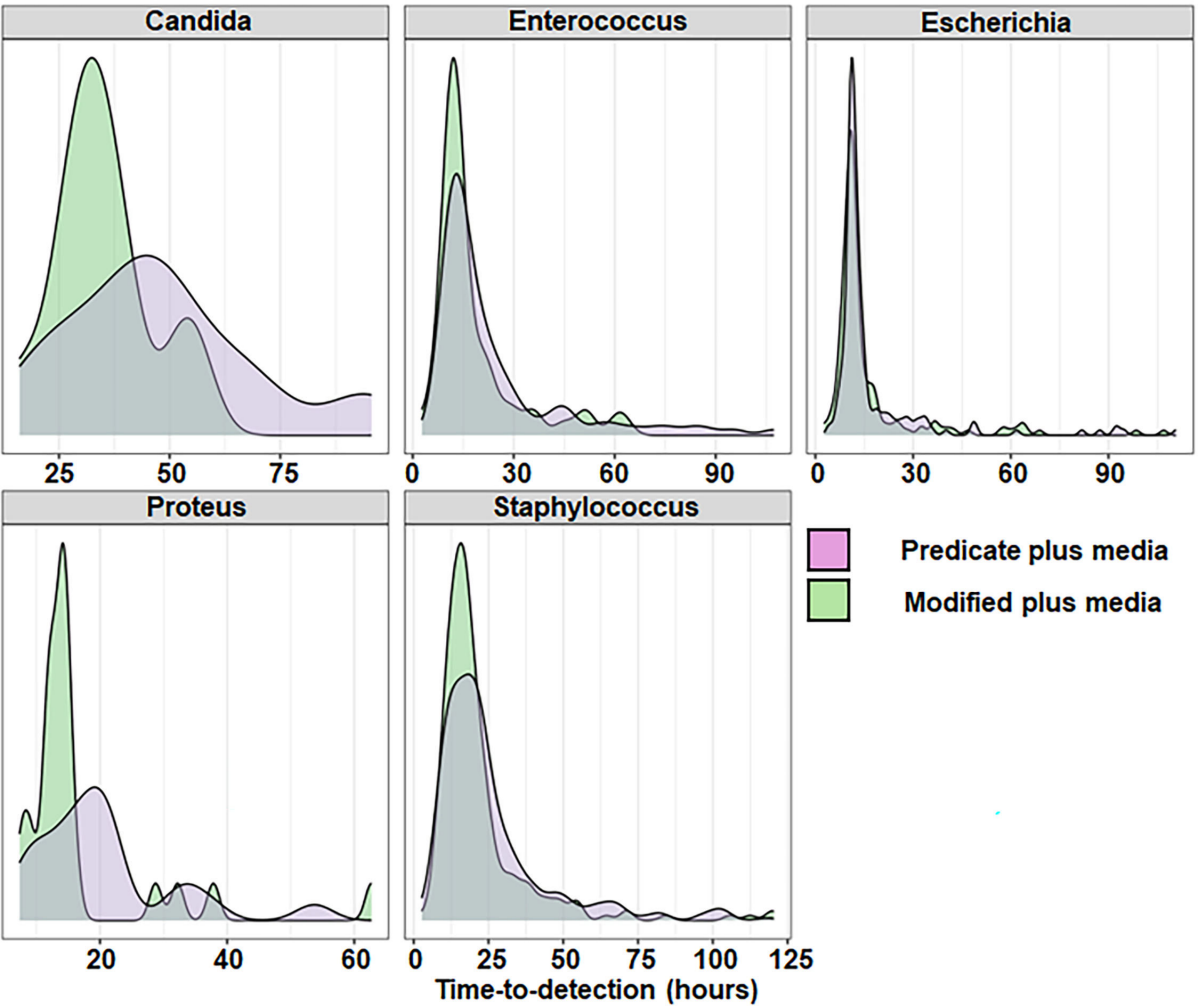
Distribution of time-to-detection (hours) results from incubation with either predicate plus (purple) or modified plus (green) media for five clinically relevant genera. Positive results were extracted from the EpiCenter microbiology data management system for time periods corresponding to incubation in either predicate plus (01/01/2023 to 06/30/2023) or modified plus media (10/20/2023 to 03/20/2024).

Data from clinical specimens collected from before and after the switch from predicate plus media to modified plus media were also assessed. While the primary objective was to evaluate changes in TTD, additional metrics—including average blood culture fill volume, positivity rate, and contamination rate—were also examined to ensure that the media change did not adversely affect other aspects of performance. The average volume of collected blood per bottle was 8.0 mL and 8.9 mL during the periods in which the predicate plus media and modified plus media were utilized, respectively. Although the absolute number of positives was higher in the predicate plus media group compared to the modified plus media group (1,075 versus 873 [18.8% reduction from 1,075]), positivity (7.8% and 7.6%; difference = 0.33%; *P*-value = 0.3320) rates were similar between periods that utilized predicate plus and modified plus media. In addition, no difference in contamination was observed between use of predicate plus and modified plus media (0.73% and 0.63%; difference = 0.10%; *P*-value = 0.3560) (Table S4). Overall (all organisms combined), modified plus media demonstrated a TTD of 20.3 hours compared to 23.2 hours for predicate plus media. This represented a statistically significant mean difference of −2.9 hours (*P* < 0.05; Table S5 and Fig. S1). For the 12 genera represented by the clinical isolates, modified plus media was associated with lower TTD compared to predicate plus media for *Enterococcus* (mean difference of −5.8 hours; *P* < 0.05), *Escherichia* (mean difference of 0.7 hours; *P* < 0.05), *Proteus* (mean difference of −3.5 hours; *P* < 0.05), and *Staphylococcus* (mean difference of 3.6 hours; *P* < 0.01) (Fig. 2; Table S5). The other eight genera (including *Candida*) showed differences with the modified plus media that were not significantly different from the predicate plus media (Fig. 2; Table S5). *Enterococcus faecalis* (mean difference = −5.3 hours; *P* < 0.05), *Staphylococcus epidermidis* (mean difference = −10.4 hours; *P* < 0.001), *Staphylococcus lugdunensis* (mean difference = −14.4 hours; *P* < 0.05), *Staphylococcus capitis* (mean difference = −11.2 hours; *P* < 0.05), *Escherichia coli* (mean difference = −0.8 hours; *P* < 0.05), and *Proteus mirabilis* (mean difference = −4.3 hours; *P* < 0.05) were species from their respective genera that largely drove the difference in TTD between the two media types (Table S6 and Fig. S2). An additional subgroup analysis was performed for organism groupings of clinical significance. As shown in Fig. 3 (and Table S7; also see Table S8), modified plus media led to a significantly lower TTD for organisms commonly associated with blood culture contamination (mean difference = −5.6 hours; *P* < 0.01), for coagulase-negative staphylococci (mean difference = −7.0 hours; *P* < 0.01), for organisms of high prevalence in bloodstream infections (mean difference = −2.7; *P* < 0.01), and for *Enterobacterales* (mean difference = −1.3 hours; *P* < 0.05). Results were grouped into TTD ranges of ≤24 hours, >24 hours, >48 hours, >72 hours, and >96 hours. The percentage of positive results with TTDs falling in the >24-, >48-, and >72-hour windows decreased significantly after implementation of modified plus media, with a corresponding increase in positive results falling within the 0- to 24-hour window (Table 2). Figure 4 shows the distribution of positive results over time (hours) for predicate plus and modified plus media vials for common genera associated with bloodstream infections. The increased and left-shifted mode demonstrates more positive results detected at earlier times following the implementation of modified plus media.

**TABLE 2:** Proportion of clinical organisms detected at various intervals during each study period

Time-to-detection	Predicate plus (N = 1075)	Modified plus (N = 873)	*P*-value
Within 24 hours	772 (71.8%)	682 (78.1%)	0.002
Over 24 hours	303 (28.2%)	191 (21.9%)	0.002
Over 48 hours	113 (10.5%)	60 (6.9%)	0.006
Over 72 hours	46 (4.3%)	17 (1.9%)	0.006
Over 96 hours	16 (1.5%)	10 (1.1%)	0.647

## DISCUSSION

The objective of this study was to compare the TTD of organisms in blood between predicate and modified BD BACTEC Plus Aerobic/F Culture Vials. A significant reduction in TTD was observed with the modified plus media across a range of organisms, demonstrating the ability of the modified media to achieve more rapid detection of bloodstream infections. In paired analytical specimens, modified media showed a significantly lower overall mean TTD for a majority of the genera that were tested, including *Staphylococcus*, *Escherichia*, *Klebsiella*, *and Enterococcus*. This was also the case for Gram-negative and Gram-positive groups. Notably, yeasts (including *C. albicans*, *C. glabrata*, and *C. parapsilosis*) displayed a substantially lower TTD, with a mean difference of −12.8 hours (*P* < 0.001) when modified plus media was utilized. While such large improvements were observed in TTD for the yeast genera, a statistically significant difference was not observed with clinical specimens, largely due to a small sample size at the study site. Overall, 20 of 32 individual organisms tested had significantly lower TTD with modified plus media. Consistent with analytical testing, clinical specimens demonstrated a significant reduction in overall TTD (20.3 hours vs. 23.2 hours; mean difference of 2.9 hours, *P* < 0.05). A lower TTD for modified media was also observed across several genera, including *Enterococcus*, *Escherichia*, *Proteus*, and *Staphylococcus*. Clinically relevant species, such as *Escherichia coli*, *Enterococcus faecalis*, and *Proteus mirabilis*, all had lower TTDs. Importantly, no increase in TTD was associated with incubation using modified plus media.

Subgroup analysis revealed that the modified plus media significantly reduced TTD for organisms commonly associated with blood culture contamination, such as coagulase-negative staphylococci, which can also be associated with nosocomial infections and BSI due to endocarditis (21).

Contamination during blood culture can lead to false-positive results, inappropriate use of antibiotics, prolonged hospital stays, increased healthcare expense, and inappropriate clinical management, making the reduction in TTD for coagulase-negative organisms particularly important (22–29). Furthermore, coagulase-negative staphylococci are a major cause of nosocomial infections and can lead to serious clinical conditions, such as sepsis and endocarditis, and are a significant cause of infections associated with implanted medical devices (21, 30, 31). Differentiating between true infection and contamination is particularly challenging with coagulase-negative staphylococci, as best practice indicates they must be present in multiple bottles to be considered a true infection (32). Improved TTD can help resolve these questions sooner, reducing the likelihood of misclassification and ensuring appropriate clinical management (33).

Subgroup analysis also revealed that the modified plus media significantly reduced TTD for *Enterobacterales* and other high-prevalence organisms (Fig. 3; Table S7 and Table S8). These organisms are common pathogens involved in nosocomial infections and severe conditions such as sepsis. Several organisms classified as high prevalence can also develop antibiotic resistance, which exemplifies why a shortened TTD for these organisms can have a significant impact on targeted therapies and overall patient care (34–37). The use of the modified media resulted in a significantly reduced TTD for “high prevalence” (Fig. 3; according to those identified by the BD Insights Research Database; Becton, Dickinson and Company) organisms that are commonly encountered in the clinical laboratory. Therefore, the introduction of modified plus media in routine blood cultures should help improve detection of and treatment for bloodstream infections. Yeast organisms, such as *Candida* species, have become increasingly prevalent in recent years and are significant contributors to bloodstream infections. In particular, *C. glabrata*, which grows at a relatively slow rate compared to other *Candida* species, showed the largest reduction in TTD. Rapid detection of bloodstream infections attributed to these organisms is crucial because empiric antimicrobial treatments often do not cover yeasts and molds, leading to significant delays in appropriate treatments. Delays in initiation of antifungal treatments can result in prolonged infection, increased morbidity, and high mortality rates (38).

The connection between TTD and time to treatment for blood culture is critical when managing a bloodstream infection. Reducing the time to detection and treatment can effectively improve patient outcomes, as prompt and accurate treatment can decrease infection severity, shorten hospital stays, and lower mortality rates (39–45). Faster detection and treatment can also reduce healthcare expenses by minimizing the duration of hospital stays and preventing complications associated with bloodstream infections (41, 46). As part of automated blood culture, the modification to the BD BACTEC Plus Aerobic/F Culture Vial should reduce TTD for some organisms and impact the overall time to treatment, although this direct outcome was not measured in this study.

### Limitations

The clinical component of this study was conducted across two six-month periods at a single institution. This may limit the generalizability of the study’s findings, as patient populations and pathogen prevalence may vary in different regions of the USA. A larger, multicenter study would provide more robust data. The sample size in this study, while substantial, may not capture the full diversity of pathogens encountered in different clinical settings. In addition, the clinical data presented here involve retrospective analysis of data related to the predicate plus and modified plus media (phase 1), and systematic differences (if any) in methodology, collection staff, and laboratory procedures were not accounted for in this study. Also, no data were presented here to determine the impact of lower TTD on patient outcomes. Although the analytical data were similar for 3 mL and 10 mL volumes, differences in volume in a real-world clinical laboratory setting could more drastically impact results compared with seeded studies. Finally, we did not include blood collections from pediatric patients, which may have introduced some spectrum bias into our results. The required minimum number of isolation occurrences to be included in the clinical data was 15. Therefore, some rarer organisms were not included in these analyses.

### Conclusions

The findings presented here suggest that implementation of modified plus media can lead to reductions in TTD for a range of organisms detected in blood culture. Of note, modified plus media increased the proportion of organisms detected within 24 and 48 hours. Additional work is required to fully characterize the impact of modified plus media on TTD for clinically significant organisms, including yeast. It will be important to determine whether shorter TTD, obtained following the implementation of modified plus media, translates into faster diagnosis and treatment of bloodstream infections and facilitates improved patient outcomes.

## Data Availability

Source data will be made available upon request to the corresponding author.

## SUPPLEMENTAL MATERIAL

Supplemental material spectrum.01969-25-s0001.docx Tables S1 to S8; Fig. S1 and S2.

ASM does not own the copyrights to Supplemental Material that may be linked to, or accessed through, an article. The authors have granted ASM a non-exclusive, world-wide license to publish the Supplemental Material files. Please contact the corresponding author directly for reuse.

## References

[1] Leibovici L, Samra Z, Konigsberger H, Drucker M, Ashkenazi S, Pitlik SD. 1995. Long-term survival following bacteremia or fungemia. JAMA 274:807–812. doi:10.1001/jama.1995.03530100047033 7650804

[2] Bassetti M, Righi E, Carnelutti A. 2016. Bloodstream infections in the intensive care unit. Virulence 7:267–279. doi:10.1080/21505594.2015.1134072 26760527 PMC4871677

[3] Wang L, Zhang L, Huang X, Xu H, Huang W. 2024. Bloodstream infection clusters for critically ill patients: analysis of two-center retrospective cohorts. BMC Infect Dis 24:306. doi:10.1186/s12879-024-09203-5 38481153 PMC10935929

[4] Timsit J-F, Ruppé E, Barbier F, Tabah A, Bassetti M. 2020. Bloodstream infections in critically ill patients: an expert statement. Intensive Care Med 46:266–284. doi:10.1007/s00134-020-05950-6 32047941 PMC7223992

[5] Rudd KE, Johnson SC, Agesa KM, Shackelford KA, Tsoi D, Kievlan DR, Colombara DV, Ikuta KS, Kissoon N, Finfer S, Fleischmann-Struzek C, Machado FR, Reinhart KK, Rowan K, Seymour CW, Watson RS, West TE, Marinho F, Hay SI, Lozano R, Lopez AD, Angus DC, Murray CJL, Naghavi M. 2020. Global, regional, and national sepsis incidence and mortality, 1990-2017: analysis for the global burden of disease study. Lancet 395:200–211. doi:10.1016/S0140-6736(19)32989-7 31954465 PMC6970225

[6] Diekema DJ, Beekmann SE, Chapin KC, Morel KA, Munson E, Doern GV. 2003. Epidemiology and outcome of nosocomial and community-onset bloodstream infection. J Clin Microbiol 41:3655–3660. doi:10.1128/JCM.41.8.3655-3660.2003 12904371 PMC179863

[7] Weinstein MP, Reller LB, Murphy JR, Lichtenstein KA. 1983. The clinical significance of positive blood cultures: a comprehensive analysis of 500 episodes of bacteremia and fungemia in adults. I. laboratory and epidemiologic observations. Clin Infect Dis 5:35–53. doi:10.1093/clinids/5.1.35 6828811

[8] Weinstein MP, Murphy JR, Reller LB, Lichtenstein KA. 1983. The clinical significance of positive blood cultures: a comprehensive analysis of 500 episodes of bacteremia and fungemia in adults II. clinical observations, with special reference to factors influencing prognosis. Clin Infect Dis 5:54–70. doi:10.1093/clinids/5.1.54 6828812

[9] Bates DW, Pruess KE, Lee TH. 1995. How bad are bacteremia and sepsis? Outcomes in a cohort with suspected bacteremia. Arch Intern Med 155:593–598. doi:10.1001/archinte.1995.00430060050006 7887754

[10] Schechner V, Wulffhart L, Temkin E, Feldman SF, Nutman A, Shitrit P, Schwaber MJ, Carmeli Y. 2022. One-year mortality and years of potential life lost following bloodstream infection among adults: a nation-wide population based study. Lancet Reg Health Eur 23:100511. doi:10.1016/j.lanepe.2022.100511 36158527 PMC9490098

[11] Dunagan WmC, Woodward RS, Medoff G, Gray JL III, Casabar E, Smith MD, Lawrenz CA, Spitznagel E. 1989. Antimicrobial misuse in patients with positive blood cultures. Am J Med 87:253–259. doi:10.1016/S0002-9343(89)80146-9 2773963

[12] Ispahani P, Pearson NJ, Greenwood D. 1987. An analysis of community and hospital-acquired bacteraemia in a large teaching hospital in the United Kingdom. Q J Med 63:427–440. doi:10.1093/oxfordjournals.qjmed.a068113 3310074

[13] von Laer A, N’Guessan MA, Touré FS, Nowak K, Groeschner K, Ignatius R, Friesen J, Tomczyk S, Leendertz FH, Eckmanns T, Akoua-Koffi C. 2021. Implementation of automated blood culture with quality assurance in a resource-limited setting. Front Med 8:627513. doi:10.3389/fmed.2021.627513 PMC8176090 34095162

[14] Halperin AV, Del Castillo Polo JA, Cortes-Cuevas JL, Cardenas Isasi MJ, Ampuero Morisaki M, Birch R, Sánchez Díaz AM, Cantón R. 2022. Impact of automated blood culture systems on the management of bloodstream infections: results from a crossover diagnostic clinical trial. Microbiol Spectr 10:e0143622. doi:10.1128/spectrum.01436-22 36094318 PMC9602854

[15] Samuel L. 2023. Direct-from-blood detection of pathogens: a review of technology and challenges. J Clin Microbiol 61:e0023121. doi:10.1128/jcm.00231-21 37222587 PMC10358183

[16] Pliakos EE, Andreatos N, Shehadeh F, Ziakas PD, Mylonakis E. 2018. The cost-effectiveness of rapid diagnostic testing for the diagnosis of bloodstream infections with or without antimicrobial stewardship. Clin Microbiol Rev 31:e00095-17. doi:10.1128/CMR.00095-17 29848775 PMC6056844

[17] Reimer LG, Wilson ML, Weinstein MP. 1997. Update on detection of bacteremia and fungemia. Clin Microbiol Rev 10:444–465. doi:10.1128/CMR.10.3.444 9227861 PMC172929

[18] Babu JP, Schell RF, Le Frock JL. 1978. Evaluation of twenty-three blood culture media. J Clin Microbiol 8:288–292. doi:10.1128/jcm.8.3.288-292.1978 701464 PMC275233

[19] Loonen AJM, Wolffs PFG, Bruggeman CA, van den Brule AJC. 2014. Developments for improved diagnosis of bacterial bloodstream infections. Eur J Clin Microbiol Infect Dis 33:1687–1702. doi:10.1007/s10096-014-2153-4 24848132

[20] Kadri SS, Lai YL, Warner S, Strich JR, Babiker A, Ricotta EE, Demirkale CY, Dekker JP, Palmore TN, Rhee C, Klompas M, Hooper DC, Powers JH 3rd, Srinivasan A, Danner RL, Adjemian J. 2021. Inappropriate empirical antibiotic therapy for bloodstream infections based on discordant in-vitro susceptibilities: a retrospective cohort analysis of prevalence, predictors, and mortality risk in US hospitals. Lancet Infect Dis 21:241–251. doi:10.1016/S1473-3099(20)30477-1 32916100 PMC7855478

[21] Becker K, Heilmann C, Peters G. 2014. Coagulase-negative staphylococci. Clin Microbiol Rev 27:870–926. doi:10.1128/CMR.00109-13 25278577 PMC4187637

[22] Long B, Koyfman A. 2016. Best clinical practice: blood culture utility in the emergency department. J Emerg Med 51:529–539. doi:10.1016/j.jemermed.2016.07.003 27639424

[23] Hughes JA, Cabilan CJ, Williams J, Ray M, Coyer F. 2018. The effectiveness of interventions to reduce peripheral blood culture contamination in acute care: a systematic review protocol. Syst Rev 7:216. doi:10.1186/s13643-018-0877-4 30497526 PMC6267024

[24] Doern GV, Carroll KC, Diekema DJ, Garey KW, Rupp ME, Weinstein MP, Sexton DJ. 2019. Practical guidance for clinical microbiology laboratories: a comprehensive update on the problem of blood culture contamination and a discussion of methods for addressing the problem. Clin Microbiol Rev 33:00009–00019. doi:10.1128/CMR.00009-19 PMC6822992 31666280

[25] Geisler BP, Jilg N, Patton RG, Pietzsch JB. 2019. Model to evaluate the impact of hospital-based interventions targeting false-positive blood cultures on economic and clinical outcomes. J Hosp Infect 102:438–444. doi:10.1016/j.jhin.2019.03.012 30928573

[26] Gander RM, Byrd L, DeCrescenzo M, Hirany S, Bowen M, Baughman J. 2009. Impact of blood cultures drawn by phlebotomy on contamination rates and health care costs in a hospital emergency department. J Clin Microbiol 47:1021–1024. doi:10.1128/JCM.02162-08 19171686 PMC2668314

[27] Hall KK, Lyman JA. 2006. Updated review of blood culture contamination. Clin Microbiol Rev 19:788–802. doi:10.1128/CMR.00062-05 17041144 PMC1592696

[28] Weinstein MP. 2003. Blood culture contamination: persisting problems and partial progress. J Clin Microbiol 41:2275–2278. doi:10.1128/JCM.41.6.2275-2278.2003 12791835 PMC156489

[29] Schifman RB, Strand CL, Meier FA, Howanitz PJ. 1998. Blood culture contamination: a College of American Pathologists Q-Probes study involving 640 institutions and 497134 specimens from adult patients. Arch Pathol Lab Med 122:216–221. 9823858

[30] Noshak MA, Rezaee MA, Hasani A, Mirzaii M. 2020. The role of the coagulase-negative Staphylococci (CoNS) in infective endocarditis; a narrative review from 2000 to 2020. CPB 21:1140–1153. doi:10.2174/1389201021666200423110359 32324510

[31] Michels R, Last K, Becker SL, Papan C. 2021. Update on coagulase-negative Staphylococci-what the clinician should know. Microorganisms 9:doi. doi:10.3390/microorganisms9040830 PMC8070739 33919781

[32] Anonymous, CLSI. 2022. Principles and procedures for blood cultures. 2nd ed. Clinical and Laboratory Standards Institute.

[33] Kassis C, Rangaraj G, Jiang Y, Hachem RY, Raad I. 2009. Differentiating culture samples representing coagulase-negative staphylococcal bacteremia from those representing contamination by use of time-to-positivity and quantitative blood culture methods. J Clin Microbiol 47:3255–3260. doi:10.1128/JCM.01045-09 19692564 PMC2756952

[34] Lee SY, Kotapati S, Kuti JL, Nightingale CH, Nicolau DP. 2006. Impact of extended-spectrum beta-lactamase-producing Escherichia coli and Klebsiella species on clinical outcomes and hospital costs: a matched cohort study. Infect Control Hosp Epidemiol 27:1226–1232. doi:10.1086/507962 17080381

[35] Kollef MH. 1999. Antimicrobial therapy of ventilator-associated pneumonia: how to select an appropriate drug regimen. Chest 115:8–11. doi:10.1378/chest.115.1.8 9925056

[36] Gupta V, Ye G, Olesky M, Lawrence K, Murray J, Yu K. 2019. Trends in resistant Enterobacteriaceae and Acinetobacter species in hospitalized patients in the United States: 2013-2017. BMC Infect Dis 19:742. doi:10.1186/s12879-019-4387-3 31443635 PMC6708167

[37] Zilberberg MD, Nathanson BH, Sulham K, Fan W, Shorr AF. 2017. Carbapenem resistance, inappropriate empiric treatment and outcomes among patients hospitalized with Enterobacteriaceae urinary tract infection, pneumonia and sepsis. BMC Infect Dis 17:279. doi:10.1186/s12879-017-2383-z 28415969 PMC5393012

[38] Fernandez J, Erstad BL, Petty W, Nix DE. 2009. Time to positive culture and identification for Candida blood stream infections. Diagn Microbiol Infect Dis 64:402–407. doi:10.1016/j.diagmicrobio.2009.04.002 19446982

[39] Puro V, Coppola N, Frasca A, Gentile I, Luzzaro F, Peghetti A, Sganga G. 2022. Pillars for prevention and control of healthcare-associated infections: an Italian expert opinion statement. Antimicrob Resist Infect Control 11:87. doi:10.1186/s13756-022-01125-8 35725502 PMC9207866

[40] Odada D, Munyi H, Gatuiku J, Thuku R, Nyandigisi J, Wangui A, Ashihundu E, Nyakiringa B, Kimeu J, Musumbi M, Adam RD. 2023. Reducing the rate of central line-associated bloodstream infections; a quality improvement project. BMC Infect Dis 23:745. doi:10.1186/s12879-023-08744-5 37904103 PMC10617146

[41] Sartelli M, Marini CP, McNelis J, Coccolini F, Rizzo C, Labricciosa FM, Petrone P. 2024. Preventing and controlling healthcare-associated infections: the first principle of every antimicrobial stewardship program in hospital settings. Antibiotics (Basel) 13:896. doi:10.3390/antibiotics13090896 39335069 PMC11428707

[42] Tabak YP, Vankeepuram L, Ye G, Jeffers K, Gupta V, Murray PR. 2018. Blood culture turnaround time in U.S. acute care hospitals and implications for laboratory process optimization. J Clin Microbiol 56:00500–00518. doi:10.1128/JCM.00500-18 PMC6258864 30135230

[43] Costa SP, Carvalho CM. 2022. Burden of bacterial bloodstream infections and recent advances for diagnosis. Pathog Dis 80:ftac027. doi:10.1093/femspd/ftac027 35790126

[44] Sautter RL, Parrott JS, Nachamkin I, Diel C, Tom RJ, Bobenchik AM, Bradford JY, Gilligan P, Halstead DC, LaSala PR, Mochon AB, Mortensen JE, Boyce L, Baselski V. 2024. American Society for Microbiology evidence-based laboratory medicine practice guidelines to reduce blood culture contamination rates: a systematic review and meta-analysis. Clin Microbiol Rev 37:e0008724. doi:10.1128/cmr.00087-24 39495314 PMC11629619

[45] Qin Y, Liao Y, Zhou J, Liu W, Chen H, Chen X, Wang W, Zhang N, Zhao Y, Wang L, Gu B, Liu S. 2025. Comparative evaluation of BacT/ALERT VIRTUO and BACTEC FX400 blood culture systems for the detection of bloodstream infections. Microbiol Spectr 13:e0185024. doi:10.1128/spectrum.01850-24 39611835 PMC11705859

[46] Su LH, Chen IL, Tang YF, Lee JS, Liu JW. 2020. Increased financial burdens and lengths of stay in patients with healthcare-associated infections due to multidrug-resistant bacteria in intensive care units: a propensity-matched case-control study. PLoS One 15:e0233265. doi:10.1371/journal.pone.0233265 32421700 PMC7233534

