# Supplementary material for: Improved time-to-detection with the new formulation of BD BACTEC Plus Aerobic/F Culture Vials: a real-world evidence study
Source: Microbiol Spectr. 2025 Sep 12;13(10):e01969-25. doi: 10.1128/spectrum.01969-25 (PMC12502807; doi:10.1128/spectrum.01969-25)

# Improved Time-to-Detection with the New Formulation of BD BACTEC™ Plus Aerobic/F Culture Vials: A Real-World Evidence study

## Kayla Van Benten^1#^, Mondraya Howard^2^, Valentin Parvu^1^, Chris Massey^1^ , Stephanie Frey^1^

^1^ Becton, Dickinson and Company, Diagnostic Solutions, 7 Loveton Circle, Sparks, MD, USA

^2^Penn Medicine Lancaster General Health, Laboratory Department, Lancaster, USA

# SUPPLEMENTAL MATERIAL

## **TABLE S1**

| **Table S1.** Comparison of predicate and modified plus media in analytical testing; time-to-detection (hours) results by species; blood volume tested = 10 ml | | | | | |
| --- | --- | --- | --- | --- | --- |
| **Category** | **Media type** | **N** | **Mean (SD)** | **Median (25%, 75%)** | **p-value** |
| ***Acinetobacter baumannii*** | Predicate plus | 5 | 9.3 (0.1) | 9.3 (9.2, 9.3) | 0.181 |
|  | Modified plus | 5 | 9.3 (0.0) | 9.3 (9.3, 9.3) |  |
| ***Candida albicans*** | Predicate plus | 20 | 28.9 (2.6) | 28.6 (26.9, 30.1) | <0.001 |
|  | Modified plus | 20 | 25.8 (2.2) | 25.6 (24.6 26.6) |  |
| ***Candida glabrata*** | Predicate plus | 20 | 56.3 (14.7) | 48.2 (46.9, 71.2) | <0.001 |
|  | Modified plus | 20 | 27.8 (3.2) | 26.4 (25.7, 30.8) |  |
| ***Candida parapsilosis*** | Predicate plus | 5 | 31.7 (1.0) | 31.8 (30.8, 32.6) | <0.01 |
|  | Modified plus | 5 | 29.7 (1.0) | 30.0 (28.8, 30.3) |  |
| ***Candida tropicalis*** | Predicate plus | 5 | 16.9 (0.3) | 17.1 (16.6, 17.1) | 0.327 |
|  | Modified plus | 5 | 17.4 (0.8) | 17.1 (16.8, 18.1) |  |
| ***Enterobacter cloacae*** | Predicate plus | 5 | 10.2 (0.2) | 10.1 (10.1, 10.3) | 0.576 |
|  | Modified plus | 5 | 10.3 (0.2) | 10.1 (10.1, 10.5) |  |
| ***Enterococcus avium*** | Predicate plus | 5 | 16.8 (0.3) | 16.8 (16.5, 17.1) | <0.001 |
|  | Modified plus | 5 | 15.2 (0.4) | 15.1 (14.9, 15.5) |  |
| ***Enterococcus faecalis*** | Predicate plus | 20 | 11.3 (0.4) | 11.2 (11.1, 11.5) | 0.123 |
|  | Modified plus | 20 | 11.2 (0.3) | 11.1 (10.9, 11.5) |  |
| ***Enterococcus faecium*** | Predicate plus | 5 | 17.3 (0.4) | 17.3 (16.8, 17.6) | <0.001 |
|  | Modified plus | 5 | 13.8 (0.1) | 13.8 (13.7, 13.9) |  |
| ***Escherichia coli*** | Predicate plus | 95 | 11.0 (1.8) | 10.4 (9.6, 11.8) | <0.001 |
|  | Modified plus | 95 | 9.6 (0.3) | 9.6 (9.3, 9.9) |  |
| ***Klebsiella aerogenes*** | Predicate plus | 5 | 12.4 (0.2) | 12.4 (12.3, 12.6) | <0.001 |
|  | Modified plus | 5 | 10.7 (0.2) | 10.8 (10.5, 10.9) |  |
| ***Klebsiella oxytoca*** | Predicate plus | 5 | 11.0 (0.1) | 10.9 (10.8, 11.1) | 0.617 |
|  | Modified plus | 5 | 10.9 (0.2) | 10.9 (10.7, 11.1) |  |
| ***Klebsiella pneumoniae*** | Predicate plus | 30 | 10.8 (0.8) | 10.7 (10.1, 11.5) | <0.001 |
|  | Modified plus | 30 | 10.3 (0.6) | 10.3 (9.7, 10.6) |  |
| ***Morganella morganii*** | Predicate plus | 5 | 11.9 (0.4) | 11.9 (11.6, 12.2) | <0.01 |
|  | Modified plus | 5 | 11.2 (0.1) | 11.2 (11.1, 11.2) |  |
| ***Proteus mirabilis*** | Predicate plus | 10 | 16.8 (1.3) | 16.8 (15.6, 17.7) | <0.001 |
|  | Modified plus | 10 | 11.3 (0.4) | 11.2 (10.9, 11.7) |  |
| ***Pseudomonas aeruginosa*** | Predicate plus | 15 | 16.9 (0.5) | 16.7 (16.4, 17.3) | 0.203 |
|  | Modified plus | 15 | 17.1 (0.6) | 17.1 (16.4, 17.6) |  |
| ***Serratia marcescens*** | Predicate plus | 5 | 12.2 (0.1) | 12.3 (12.1, 12.3) | 0.190 |
|  | Modified plus | 5 | 12.4 (0.2) | 12.5 (12.2, 12.5) |  |
| ***Staphylococcus aureus*** | Predicate plus | 95 | 12.5 (1.6) | 11.9 (11.6, 12.6) | <0.05 |
|  | Modified plus | 95 | 12.4 (1.3) | 12.0 (11.6, 12.7) |  |
| ***Staphylococcus capitis*** | Predicate plus | 5 | 28.6 (1.7) | 28.3 (27.3, 30.1) | <0.05 |
|  | Modified plus | 5 | 25.4 (0.6) | 25.3 (24.9, 25.9) |  |
| ***Staphylococcus epidermidis*** | Predicate plus | 45 | 17.4 (1.0) | 17.6 (16.8, 18.2) | <0.001 |
|  | Modified plus | 45 | 16.1 (1.0) | 15.7 (15.4, 16.8) |  |
| ***Staphylococcus haemolyticus*** | Predicate plus | 5 | 14.1 (0.2) | 13.9 (13.9, 14.3) | <0.01 |
|  | Modified plus | 5 | 13.3 (0.3) | 13.4 (13.0, 13.7) |  |
| ***Staphylococcus hominis***  ***ssp hominis*** | Predicate plus | 20 | 26.4 (10.1) | 21.6 (21.0, 31.5) | <0.001 |
|  | Modified plus | 20 | 14.8 (0.7) | 14.6 (14.2, 15.4) |  |
| ***Staphylococcus lugdunensis*** | Predicate plus | 5 | 16.7 (0.5) | 16.9 (16.2, 17.1) | <0.05 |
|  | Modified plus | 5 | 16.0 (0.2) | 15.9 (15.8, 16.1) |  |
| ***Staphylococcus saprophyticus*** | Predicate plus | 10 | 19.1 (2.0) | 18.9 (17.5, 20.0) | <0.001 |
|  | Modified plus | 10 | 14.6 (0.3) | 14.7 (14.3, 14.9) |  |
| ***Staphylococcus warneri*** | Predicate plus | 5 | 17.6 (0.6) | 17.6 (17.1, 18.2) | <0.001 |
|  | Modified plus | 5 | 14.1 (0.3) | 14.1 (13.8, 14.3) |  |
| ***Streptococcus agalactiae***  **(Strep. group B)** | Predicate plus | 10 | 8.6 (0.2) | 8.6 (8.6, 8.7) | <0.01 |
|  | Modified plus | 10 | 8.3 (0.2) | 8.2 (8.2, 8.6) |  |
| ***Streptococcus anginosus*** | Predicate plus | 5 | 13.9 (0.2) | 13.9 (13.8, 14.1) | 0.429 |
|  | Modified plus | 5 | 14.0 (0.3) | 14.1 (13.8, 14.3) |  |
| ***Streptococcus mitis*** | Predicate plus | 5 | 11.3 (0.3) | 11.4 (11.1, 11.5) | 0.457 |
|  | Modified plus | 5 | 11.6 (0.6) | 11.6 (11.0, 12.2) |  |
| ***Streptococcus oralis*** | Predicate plus | 5 | 11.9 (0.2) | 11.8 (11.7, 12.1) | 0.308 |
|  | Modified plus | 5 | 12.0 (0.3) | 12.1 (11.7, 12.2) |  |
| ***Streptococcus pneumoniae*** | Predicate plus | 15 | 11.5 (0.5) | 11.3 (11.2, 11.9) | 0.939 |
|  | Modified plus | 15 | 11.5 (0.3) | 11.6 (11.2, 11.8) |  |
| ***Streptococcus pyogenes***  **(Strep. group A)** | Predicate plus | 5 | 10.2 (0.1) | 10.2 (10.1, 10.3) | 0.995 |
|  | Modified plus | 5 | 10.2 (0.4) | 10.0 (9.9, 10.6) |  |
| ***Streptococcus salivarius*** | Predicate plus | 5 | 10.4 (0.2) | 10.2 (10.2, 10.7) | <0.05 |
|  | Modified plus | 5 | 10.1 (0.2) | 10.2 (10.0, 10.2) |  |

## **TABLE S2**

| **Table S2.** Comparison of predicate and modified plus media in analytical testing; results by genera, yeast, and Gram stain category; blood volume tested = 3 ml | | | | | | |
| --- | --- | --- | --- | --- | --- | --- |
| **Category** | **Media type** | **N** | **Mean (SD)** | **Median (25%, 75%)** | **p-value** |  |
| **Overall** | Predicate plus | 500 | 16.2 (10.4) | 12.2 (11.0, 17.2) | <0.001 |  |
|  | Modified plus | 500 | 13.6 (5.1) | 11.8 (10.1, 15.3) |  |  |
| ***Acinetobacter*** | Predicate plus | 5 | 9.3 (0.1) | 9.3 (9.2, 9.3) | 0.181 |  |
|  | Modified plus | 5 | 9.3 (0.0) | 9.3 (9.3, 9.3) |  |  |
| ***Enterobacter*** | Predicate plus | 5 | 10.2 (0.2) | 10.1 (10.1, 10.3) | 0.576 |  |
|  | Modified plus | 5 | 10.3 (0.2) | 10.1 (10.1, 10.5) |  |  |
| ***Enterococcus*** | Predicate plus | 30 | 13.2 (2.8) | 11.5 (11.1, 16.7) | <0.001 |  |
|  | Modified plus | 30 | 12.3 (1.6) | 11.4 (11.1, 13.8) |  |  |
| ***Escherichia*** | Predicate plus | 95 | 11.0 (1.8) | 10.4 (9.6, 11.8) | <0.001 |  |
|  | Modified plus | 95 | 9.6 (0.3) | 9.6 (9.3, 9.9) |  |  |
| ***Klebsiella*** | Predicate plus | 40 | 11.0 (0.9) | 10.9 (10.3, 11.7) | <0.001 |  |
|  | Modified plus | 40 | 10.4 (0.6) | 10.5 (10.0, 10.9) |  |  |
| ***Morganella*** | Predicate plus | 5 | 11.9 (0.4) | 11.9 (11.6, 12.2) | <0.01 |  |
|  | Modified plus | 5 | 11.2 (0.1) | 11.2 (11.1, 11.2) |  |  |
| ***Proteus*** | Predicate plus | 10 | 16.8 (1.3) | 16.8 (15.6, 17.7) | <.0001 |  |
|  | Modified plus | 10 | 11.3 (0.4) | 11.2 (10.9, 11.7) |  |  |
| ***Pseudomonas*** | Predicate plus | 15 | 16.9 (0.5) | 16.7 (16.4, 17.3) | 0.203 |  |
|  | Modified plus | 15 | 17.1 (0.6) | 17.1 (16.4, 17.6) |  |  |
| ***Serratia*** | Predicate plus | 5 | 12.2 (0.1) | 12.3 (12.1, 12.3) | 0.190 |  |
|  | Modified plus | 5 | 12.4 ()0.2) | 12.5 (12.2, 12.5) |  |  |
| ***Staphylococcus*** | Predicate plus | 190 | 16.2 (5.9) | 15.9 (11.9, 18.1) | <0.001 |  |
|  | Modified plus | 190 | 14.2 (2.7) | 14.1 (11.9, 15.6) |  |  |
| ***Streptococcus*** | Predicate plus | 50 | 11.0 (1.5) | 11.2 (10.2, 11.9) | 0.493 |  |
|  | Modified plus | 50 | 10.9 (1.7) | 11.2 (10.0, 11.8) |  |  |
| **Yeast** | Predicate plus | 50 | 39.0 (17.5) | 31.7 (28.1, 48.1) | <0.001 |  |
|  | Modified plus | 50 | 26.1 (4.0) | 26.1 (24.6, 27.3) |  |  |
| **Gram negative** | Predicate plus | 180 | 11.8 (2.5) | 10.9 (10.0, 12.6) | <0.001 |  |
|  | Modified plus | 180 | 10.6 (2.1) | 9.9 (9.4, 10.8) |  |  |
| **Gram positive** | Predicate plus | 270 | 14.9 (5.5) | 12.6 (11.5, 17.3) | <0.001 |  |
|  | Modified plus | 270 | 13.3 (2.7) | 12.5 (11.6, 15.1) |  |  |

## **TABLE S3**

| **Table S3.** Comparison of predicate and modified plus media in analytical testing; time-to-detection (hours) results by species; blood volume tested = 3 ml | | | | | |
| --- | --- | --- | --- | --- | --- |
| **Category** | **Media type** | **N** | **Mean (SD)** | **Median (25%, 75%)** | **p-value** |
| Overall | Predicate plus | 500 | 15.4 (9.1) | 11.9 (9.9, 16.8) | <0.001 |
|  | Modified plus | 500 | 13.8 (5.3) | 11.9 (9.8, 15.5) |  |
| ***Acinetobacter baumannii*** | Predicate plus | 5 | 8.9 (0.3) | 8.8 (8.7, 9.2) | 0.089 |
|  | Modified plus | 5 | 8.6 (0.1) | 8.5 (8.5, 8.7) |  |
| ***Candida albicans*** | Predicate plus | 20 | 28.4 (3.1) | 29.4 (25.5, 30.5) | <0.001 |
|  | Modified plus | 20 | 26.2 (2.1) | 26.2 (25.0, 27.8) |  |
| ***Candida glabrata*** | Predicate plus | 20 | 51.4 (4.2) | 50.5 (48.0, 55.2) | <0.001 |
|  | Modified plus | 20 | 26.6 (2.4) | 25.9 (24.7, 28.9) |  |
| ***Candida parapsilosis*** | Predicate plus | 5 | 33.3 (1.0) | 33.6 (32.6, 33.9) | <0.001 |
|  | Modified plus | 5 | 30.6 (0.6) | 30.6 (30.1, 31.1) |  |
| ***Candida tropicalis*** | Predicate plus | 5 | 18.4 (0.8) | 18.1 (17.9, 19.1) | <0.05 |
|  | Modified plus | 5 | 17.1 (0.4) | 17.1 (16.8, 17.3) |  |
| ***Enterobacter cloacae*** | Predicate plus | 5 | 9.7 (0.1) | 9.6 (9.6, 9.7) | 0.072 |
|  | Modified plus | 5 | 9.8 (0.1) | 9.8 (9.6, 9.9) |  |
| ***Enterococcus avium*** | Predicate plus | 5 | 15.4 (0.3) | 15.1 (15.1, 15.7) | 0.997 |
|  | Modified plus | 5 | 15.4 (0.3) | 15.3 (15.0, 15.7) |  |
| ***Enterococcus faecalis*** | Predicate plus | 20 | 11.0 (0.5) | 10.9 (10.6, 11.4) | 0.157 |
|  | Modified plus | 20 | 11.1 (0.5) | 11.0 (10.8, 11.7) |  |
| ***Enterococcus faecium*** | Predicate plus | 5 | 13.7 (0.4) | 13.7 (13.4, 14.1) | 0.583 |
|  | Modified plus | 5 | 13.7 (0.3) | 13.6 (13.4, 14.0) |  |
| ***Escherichia coli*** | Predicate plus | 95 | 9.4 (0.4) | 9.4 (9.1, 9.7) | <0.05 |
|  | Modified plus | 95 | 9.3 (0.3) | 9.3 (9.1, 9.6) |  |
| ***Klebsiella aerogenes*** | Predicate plus | 5 | 11.1 (0.3) | 11.3 (10.8, 11.4) | <0.05 |
|  | Modified plus | 5 | 10.7 (0.1) | 10.6 (10.6, 10.7) |  |
| ***Klebsiella oxytoca*** | Predicate plus | 5 | 10.6 (0.2) | 10.6 (10.4, 10.8) | 0.806 |
|  | Modified plus | 5 | 10.6 (0.3) | 10.6 (10.4, 10.9) |  |
| ***Klebsiella pneumoniae*** | Predicate plus | 30 | 10.1 (0.6) | 9.9 (9.6, 10.5) | 0.217 |
|  | Modified plus | 30 | 10.0 (0.7) | 10.1 (9.4, 10.5) |  |
| ***Morganella morganii*** | Predicate plus | 5 | 10.8 (0.3) | 10.9 (10.6, 11.1) | 0.831 |
|  | Modified plus | 5 | 10.9 (0.1) | 10.9 (10.7, 11.0) |  |
| ***Proteus mirabilis*** | Predicate plus | 10 | 12.6 (0.5) | 12.6 (12.1, 13.1) | <0.001 |
|  | Modified plus | 10 | 11.1 (0.3) | 11.1 (10.7, 11.4) |  |
| ***Pseudomonas aeruginosa*** | Predicate plus | 15 | 16.3 (0.7) | 16.6 (15.8, 16.9) | <0.001 |
|  | Modified plus | 15 | 16.7 (0.7) | 17.1 (16.1, 17.4) |  |
| ***Serratia marcescens*** | Predicate plus | 5 | 10.9 (0.1) | 11.0 (10.7, 11.0) | <0.001 |
|  | Modified plus | 5 | 11.2 (0.1) | 11.1 (11.1, 11.4) |  |
| ***Staphylococcus aureus*** | Predicate plus | 95 | 12.9 (1.5) | 12.8 (11.4, 13.9) | <0.001 |
|  | Modified plus | 95 | 13.4 (1.6) | 13.6 (11.9, 14.7) |  |
| ***Staphylococcus capitis*** | Predicate plus | 5 | 28.3 (1.2) | 28.0 (27.4, 29.4) | 0.124 |
|  | Modified plus | 5 | 29.9 (1.1) | 30.1 (28.8, 30.9) |  |
| ***Staphylococcus epidermidis*** | Predicate plus | 45 | 20.9 (1.8) | 20.6 (19.5, 21.7) | <0.001 |
|  | Modified plus | 45 | 16.5 (1.6) | 16.1 (15.2, 16.9) |  |
| ***Staphylococcus haemolyticus*** | Predicate plus | 5 | 13.1 (0.1) | 13.1 (13.0, 13.2) | 0.101 |
|  | Modified plus | 5 | 13.0 (0.1) | 12.9 (12.9, 13.1) |  |
| **Staphylococcus hominis**  **ssp hominis** | Predicate plus | 20 | 17.8 (1.5) | 18.0 (17.1, 19.0) | <0.001 |
|  | Modified plus | 20 | 15.2 (0.6) | 15.3 (14.6, 15.6) |  |
| **Staphylococcus lugdunensis** | Predicate plus | 5 | 19.3 (0.5) | 19.3 (18.9, 19.8) | <0.001 |
|  | Modified plus | 5 | 15.8 (0.5) | 15.6 (15.3, 16.3) |  |
| **Staphylococcus saprophyticus** | Predicate plus | 10 | 15.4 (0.6) | 15.1 (14.9, 16.0) | <0.05 |
|  | Modified plus | 10 | 15.0 (0.3) | 15.0 (14.6, 15.3) |  |
| **Staphylococcus warneri** | Predicate plus | 5 | 13.9 (0.1) | 13.9 (13.8, 14.0) | 0.775 |
|  | Modified plus | 5 | 13.9 (0.2) | 13.8 (13.7, 14.1) |  |
| ***Streptococcus agalactiae***  **(Strep. group B)** | Predicate plus | 10 | 8.3 (0.2) | 8.3 (8.2, 8.4) | 0.739 |
|  | Modified plus | 10 | 8.2 (0.3) | 8.2 (7.9, 8.4) |  |
| ***Streptococcus anginosus*** | Predicate plus | 5 | 13.8 (0.2) | 13.8 (13.6, 14.0) | 0.239 |
|  | Modified plus | 5 | 13.6 (0.2) | 13.6 (13.4, 13.9) |  |
| ***Streptococcus mitis*** | Predicate plus | 5 | 11.7 (0.2) | 11.6 (11.6, 14.0) | <0.05 |
|  | Modified plus | 5 | 11.4 (0.2) | 11.4 (11.2, 11.4) |  |
| ***Streptococcus oralis*** | Predicate plus | 5 | 11.7 (0.1) | 11.6 (11.6, 11.9) | 0.804 |
|  | Modified plus | 5 | 11.7 (0.4) | 11.8 (11.4, 12.1) |  |
| ***Streptococcus pneumoniae*** | Predicate plus | 15 | 11.3 (0.4) | 11.2 (11.1, 11.6) | 0.076 |
|  | Modified plus | 15 | 11.5 (0.4) | 11.4 (11.1, 11.8) |  |
| ***Streptococcus agalactiae***  **(Strep. group B)** | Predicate plus | 5 | 10.0 (0.2) | 10.2 (9.8, 10.2) | 0.479 |
|  | Modified plus | 5 | 10.2 (0.2) | 10.2 (10.0, 10.3) |  |
| ***Streptococcus salivarius*** | Predicate plus | 5 | 10.1 (1.2) | 9.6 (9.4, 11.0) | 0.347 |
|  | Modified plus | 5 | 9.4 (0.2) | 9.4 (9.2, 9.7) |  |

## **TABLE S4**

| **Table S4.** Overall positivity and contamination rate for the two periods involving automated blood culture of clinical specimens | | | | |
| --- | --- | --- | --- | --- |
| **Diagnostic measure** | **Predicate plus media; % (n/N)** | **Modified plus media; % (n/N)** | **Difference** [95% CI] | **p-value^a^** |
| **Positivity rate** | 7.8% (1,075 / 13,470) | 7.6% (873 / 11,416) | 0.33% [-0.34, 1.00] | 0.3320 |
| **Contamination rate** | 0.73% (99 / 13,470) | 0.63% (72 / 11,416) | 0.10% [-0.10, 0.31] | 0.3560 |
| **^a^**Based on two proportions statistic with Fisher’s exact post-hoc testing | | | | |

## **TABLE S5**

| **Table S5.** Comparison of time-to-detection before (predicate plus media) and after introduction of modified plus media in clinical testing; time-to-detection (hours) results by genera | | | | | |
| --- | --- | --- | --- | --- | --- |
| **Genus** | **Media type** | **N** | **Mean (SD)** | **Median (25%, 75%)** | **p-value** |
| **Overall** | Predicate plus | 1075 | 23.2 (19.7) | 15.3 (11.3, 26.0) | <0.05 |
|  | Modified plus | 873 | 20.3 (16.5) | 14.6 (11.3, 22.0) |  |
| ***Candida*** | Predicate plus | 20 | 48.2 (21.6) | 46.9 (34.6, 58.0) | 0.07 |
|  | Modified plus | 12 | 34.9 (11.1) | 33.1 (28.0, 39.7) |  |
| ***Citrobacter*** | Predicate plus | 15 | 38.1 (24.1) | 30.1 (21.4, 50.5) | 0.516 |
|  | Modified plus | 4 | 29.7 (26.1) | 18.1 (14.3, 33.5) |  |
| ***Enterobacter*** | Predicate plus | 21 | 18.4 (18.4) | 12.9 (11.8, 18.0) | 0.983 |
|  | Modified plus | 10 | 17.0 (9.5) | 12.7 (11.7, 16.3) |  |
| ***Enterococcus*** | Predicate plus | 99 | 24.2 (20.3) | 15.8 (12.4, 25.9) | <0.05 |
|  | Modified plus | 55 | 18.4 (13.3) | 13.2 (11.2, 20.4) |  |
| ***Escherichia*** | Predicate plus | 187 | 17.3 (16.3) | 11.8 (10.8, 14.9) | <0.05 |
|  | Modified plus | 161 | 16.6 (15.8) | 11.4 (10.1, 15.0) |  |
| ***Haemophilus*** | Predicate plus | 6 | 33.9 (31.5) | 17.9 (11.7, 57.7) | 0.368 |
|  | Modified plus | 13 | 30.6 (8.6) | 30.8 (25.7, 36.3) |  |
| ***Klebsiella*** | Predicate plus | 73 | 20.3 (17.3) | 13.2 (11.5, 18.9) | 0.975 |
|  | Modified plus | 79 | 21.4 (19.8) | 13.2 (11.2, 19.9) |  |
| ***Proteus*** | Predicate plus | 21 | 20.4 (11.0) | 19.6 (13.9, 21.8) | <0.05 |
|  | Modified plus | 26 | 16.9 (11.7) | 14.1 (11.9, 14.7) |  |
| ***Pseudomonas*** | Predicate plus | 28 | 29.2 (19.1) | 22.0 (13.7, 39.3) | 0.636 |
|  | Modified plus | 34 | 24.0 (13.9) | 19.0 (17.0, 25.8) |  |
| ***Serratia*** | Predicate plus | 7 | 21.3 (12.9) | 19.7 (12.4, 22.6) | 0.315 |
|  | Modified plus | 10 | 15.1 (5.3) | 13.4 (11.7, 17.8) |  |
| ***Staphylococcus*** | Predicate plus | 334 | 25.6 (19.1) | 20.3 (13.7, 29.0) | <0.01 |
|  | Modified plus | 245 | 22.0 (16.9) | 17.4 (13.3, 22.9) |  |
| ***Streptococcus*** | Predicate plus | 154 | 16.7 (14.9) | 11.8 (9.8, 15.8) | 0.633 |
|  | Modified plus | 137 | 14.6 (10.2) | 12.4 (10.1, 16.3) |  |

## **TABLE S6**

| **Table S6.** Comparison of time-to-detection before (predicate plus media) and after introduction of modified plus media in clinical testing; time-to-detection (hours) results by species | | | | | |
| --- | --- | --- | --- | --- | --- |
| **Species** | **Media type** | **N** | **Mean (SD)** | **Median (25%, 75%)** | **p-value** |
| ***Enterococcus faecalis*** | Predicate plus | 95 | 23.9 (20.5) | 15.5 (12.4, 25.6) | <0.05 |
|  | Modified plus | 53 | 18.6 (13.5) | 13.2 (11.1, 20.5) |  |
| ***Enterobacter cloacae complex*** | Predicate plus | 21 | 18.4 (18.4) | 12.9 (11.8, 18.0) | 0.983 |
|  | Modified plus | 10 | 17.0 (9.5) | 12.7 (11.7, 16.3) |  |
| ***Escherichia coli*** | Predicate plus | 185 | 17.4 (16.4) | 11.9 (10.8, 14.9) | <0.05 |
|  | Modified plus | 161 | 16.6 (15.8) | 11.4 (10.1, 15.0) |  |
| ***Klebsiella oxytoca/***  ***Raoultella ornithinolytica*** | Predicate plus | 13 | 21.0 (20.2) | 14.5 (12.9, 17.1) | 0.96 |
|  | Modified plus | 13 | 26.3 (23.7) | 13.7 (11.9, 30.8) |  |
| ***Klebsiella pneumoniae*** | Predicate plus | 47 | 19.3 (16.7) | 12.8 (11.5, 18.5) | 0.819 |
|  | Modified plus | 58 | 19.5 (18.9) | 12.4 (11.0, 18.4) |  |
| ***Klebsiella variicola*** | Predicate plus | 7 | 19.1 (13.6) | 14.7 (12.6, 17.0) | 0.336 |
|  | Modified plus | 8 | 27.2 (19.0) | 19.7 (15.5, 35.9) |  |
| ***Proteus mirabilis*** | Predicate plus | 19 | 21.5 (11.0) | 19.6 (15.3, 21.9) | <0.05 |
|  | Modified plus | 23 | 17.2 (12.5) | 13.5 (11.8, 14.7) |  |
| ***Pseudomonas aeruginosa*** | Predicate plus | 25 | 29.9 (19.8) | 19.7 (13.9, 40.0) | 0.640 |
|  | Modified plus | 34 | 24.0 (13.9) | 19.0 (17.0, 25.8) |  |
| ***Staphylococcus aureus*** | Predicate plus | 189 | 22.8 (19.1) | 16.4 (11.5, 23.9) | 0.668 |
|  | Modified plus | 171 | 21.9 (19.0) | 16.6 (12.4, 21.7) |  |
| ***Staphylococcus capitis*** | Predicate plus | 9 | 29.4 (12.3) | 24.2 (22.1, 32.5) | 0.026 |
|  | Modified plus | 6 | 18.2 (5.4) | 18.9 (17.5, 20.6) |  |
| ***Staphylococcus epidermidis*** | Predicate plus | 85 | 32.7 (21.8) | 25.3 (21.0, 34.5) | <0.001 |
|  | Modified plus | 39 | 22.3 (12.3) | 19.1 (15.3, 22.7) |  |
| ***Staphylococcus hominis*** | Predicate plus | 23 | 23.0 (4.8) | 21.3 (19.9, 25.0) | 0.462 |
|  | Modified plus | 7 | 27.2 (9.4) | 28.9 (19.0, 34.0) |  |
| ***Streptococcus agalactiae***  **(Strep. group B)** | Predicate plus | 22 | 14.2 (13.5) | 10.6 (9.5, 13.4) | 0.371 |
|  | Modified plus | 10 | 12.7 (3.7) | 11.9 (10.3, 13.9) |  |
| ***Streptococcus anginosus*** | Predicate plus | 7 | 31.6 (16.8) | 30.1 (19.4, 43.2) | 0.114 |
|  | Modified plus | 9 | 19.2 (4.0) | 18.8 (17.7, 22.5) |  |
| ***Streptococcus dysgalactiae*** | Predicate plus | 15 | 20.5 (24.3) | 11.1 (9.4, 18.6) | 0.579 |
|  | Modified plus | 19 | 13.4 (10.7) | 11.4 (8.5, 13.7) |  |
| ***Streptococcus gallolyticus*** | Predicate plus | 6 | 9.7 (1.1) | 10.0 (9.2, 10.3) | 0.119 |
|  | Modified plus | 11 | 13.5 (6.5) | 10.7 (9.9, 12.6) |  |
| ***Staphylococcus lugdunensis*** | Predicate plus | 12 | 30.2 (18.6) | 25.0 (16.4, 33.8) | <0.05 |
|  | Modified plus | 4 | 15.8 (3.6) | 14.8 (13.7, 17.0) |  |
| ***Streptococcus mitis group*** | Predicate plus | 13 | 14.6 (2.7) | 14.8 (12.2, 16.5) | 0.496 |
|  | Modified plus | 12 | 15.1 (3.1) | 15.1 (13.1, 17.5) |  |
| ***Streptococcus pneumoniae*** | Predicate plus | 31 | 10.5 (2.8) | 10.9 (9.8, 12.4) | 0.700 |
|  | Modified plus | 29 | 13.3 (16.6) | 10.9 (9.6, 12.4) |  |
| ***Streptococcus pyogenes***  **(Strep. group A)** | Predicate plus | 25 | 11.2 (3.1) | 11.6 (9.6, 13.1) | 0.543 |
|  | Modified plus | 13 | 33.2 (12.2) | 33.0 (27.9, 39.6) |  |

## **TABLE S7**

| **Table S7.** Comparison of time-to-detection before (predicate plus media) and after introduction of modified plus media in clinical testing; time-to-detection (hours) results by clinical category | | | | | | |
| --- | --- | --- | --- | --- | --- | --- |
| **Category** | **Media type** | **N** | **Mean (SD)** | **Median (25%, 75%)** | **p-value** | |
| **Coag. Neg. Staph** | Predicate plus | 145 | 29.3 (18.6) | 23.4 (19.7, 31.2) | <0.01 | |
|  | Modified plus | 74 | 22.3 (10.7) | 19.1 (15.7, 25.0) |  |  |
| **Common IET^a^** | Predicate plus | 143 | 24.6 (20.1) | 16.0 (12.5, 28.2) | 0.368 | |
|  | Modified plus | 89 | 20.5 (13.7) | 16.8 (12.3, 23.7) |  |  |
| **Contaminant** | Predicate plus | 157 | 29.6 (18.2) | 24.1 (19.7, 33.0) | <0.01 | |
|  | Modified plus | 88 | 24.0 (11.3) | 21.4 (16.5, 29.7) |  |  |
| **Enterobacteriaceae** | Predicate plus | 339 | 19.4 (17.6) | 12.8 (11.0, 19.7) | <0.05 | |
|  | Modified plus | 300 | 18.1 (16.5) | 12.2 (10.4, 17.1) |  |  |
| **Highest Mortality IET** | Predicate plus | 735 | 20.9 (18.4) | 13.6 (11.0, 21.4) | 0.139 | |
|  | Modified plus | 611 | 19.1 (16.5) | 13.4 (10.9, 19.1) |  |  |
| **High prevalence** | Predicate plus | 792 | 22.2 (18.8) | 14.9 (11.3, 24.5) | <0.01 | |
|  | Modified plus | 639 | 19.5 (16.5) | 13.7 (11.0, 20.5) |  |  |
| **Non-Fermenter** | Predicate plus | 46 | 25.3 (19.5) | 16.9 (12.5, 30.9) | 0.323 | |
|  | Modified plus | 34 | 24.0 (13.9) | 19.0 (17.0, 25.8) |  |  |
| **Yeast** | Predicate plus | 22 | 46.0 (21.9) | 44.4 (29.8, 55.1) | 0.085 |  |
|  | Modified plus | 13 | 33.2 (12.2) | 33.0 (27.9, 39.6) |  |  |
| **Abbreviations:** Coag., coagulase; Neg., negative; IET, inappropriate empirical treatment | | | | | | |
|  | | | | | | |
| **^a^**Organisms identified by Kadri et al., 2021(47) et. al | | | | | | |

## **TABLE S8**

| **Table S8. Identification of organism included in ‘Common IET’ category found in Figure 3 and Table S6** | | | | | | | | |
| --- | --- | --- | --- | --- | --- | --- | --- | --- |
| **High Prevalence** | **Coag. Neg. Staph** | **Enterobacteriaceae** | **Yest** | **Non-fermenters** | **Contaminants** | **Common IET** | **Highest mortality IET** | |
| *Streptococcus pneumoniae* | *Staphylococcus capitis* | *Salmonella species* | *Candida tropicalis* | *Stenotrophomonas maltophilia* | *Micrococcus luteus* | Stenotrophomonas maltophilia | Stenotrophomonas maltophilia | Klebsiella pneumoniae |
| *Staphylococcus epidermidis* | *Staphylococcus epidermidis* | *Klebsiella variicola* | *Candida parapsilosis* | *Aeromonas caviae* | *Corynebacterium aurimucosum group* | Acinetobacter baumannii/calcoaceticus complex | Streptococcus dysgalactiae | Klebsiella oxytoca/Raoultella ornithinolytica |
| *Escherichia coli* | *Staphylococcus hominis* | *Escherichia coli* | *Candida glabrata* | *Acinetobacter baumannii/calcoaceticus complex* | *Corynebacterium striatum group* | Acinetobacter baumannii | Salmonella species | Enterococcus faecalis |
| *Staphylococcus aureus* | *Staphylococcus lugdunensis* | *Citrobacter koseri* | *Candida orthopsilosis* | *Acinetobacter baumannii* | *Corynebacterium propinquum* | Enterococcus durans | Aeromonas caviae | Enterobacter cloacae complex |
| *Staphylococcus hominis* | *Staphylococcus haemolyticus* | *Morganella morganii* | *Candida albicans* | *Roseomonas mucosa* | *Corynebacterium imitans* | Roseomonas mucosa | Acinetobacter baumannii/calcoaceticus complex | Enterococcus faecium |
| *Streptococcus pyogenes (Strep. group A)* | *Staphylococcus warneri* | *Proteus vulgaris* | *Clavispora lusitaniae* | *Pseudomonas pseudoalcaligenes* | *Rothia species* | Pseudomonas pseudoalcaligenes | Acinetobacter baumannii | Providencia rettgeri |
| *Proteus mirabilis* | *Staphylococcus caprae* | *Proteus vulgaris group* | *Pichia kudriavzevii* | *Cronobacter species* | *Staphylococcus capitis* | Enterococcus faecalis | Enterococcus durans | Pseudomonas aeruginosa |
| *Staphylococcus lugdunensis* | *Staphylococcus pettenkoferi* | *Citrobacter freundii* |  | *Pseudomonas aeruginosa* | *Staphylococcus epidermidis* | Enterococcus faecium | Roseomonas mucosa | Streptococcus agalactiae (Strep. group B) |
| *Klebsiella pneumoniae* | *Staphylococcus pasteuri* | *Proteus mirabilis* |  | *Pseudomonas species* | *Staphylococcus hominis* | Pseudomonas aeruginosa | Pseudomonas pseudoalcaligenes | Citrobacter freundii complex |
| *Klebsiella oxytoca/Raoultella ornithinolytica* | *Staphylococcus simulans* | *Klebsiella pneumoniae* |  |  | *Staphylococcus lugdunensis* | Pseudomonas species | Cronobacter species | Klebsiella aerogenes |
| *Enterococcus faecalis* | *Staphylococcus schleiferi* | *Klebsiella oxytoca/Raoultella ornithinolytica* |  |  | *Staphylococcus haemolyticus* |  | Klebsiella variicola | Serratia marcescens |
| *Candida glabrata* |  | *Enterobacter cloacae complex* |  |  | *Staphylococcus warneri* |  | Escherichia coli | Pantoea septica |
| *Enterococcus faecium* |  | *Providencia rettgeri* |  |  | *Staphylococcus caprae* |  | Citrobacter koseri | Shigella sonnei |
| *Pseudomonas aeruginosa* |  | *Citrobacter freundii complex* |  |  | *Staphylococcus pettenkoferi* |  | Staphylococcus aureus | Pseudomonas species |
| *Streptococcus agalactiae (Strep. group B)* |  | *Klebsiella aerogenes* |  |  | *Staphylococcus pasteuri* |  | Morganella morganii | Escherichia hermannii |
| *Klebsiella aerogenes* |  | *Serratia marcescens* |  |  | *Staphylococcus simulans* |  | Proteus vulgaris | Serratia fonticola |
| *Candida albicans* |  | *Pantoea septica* |  |  | ***Staphylococcus schleiferi*** |  | Proteus vulgaris group | Providencia stuartii |
| *Pseudomonas species* |  | *Shigella sonnei* |  |  |  |  | Streptococcus pyogenes (Strep. group A) | Serratia liquefaciens |
|  |  | *Escherichia hermannii* |  |  |  |  | Citrobacter freundii | Hafnia alvei |
|  |  | *Serratia fonticola* |  |  |  |  | Proteus mirabilis | Citrobacter braakii |
|  |  | *Providencia stuartii* |  |  |  |  |  |  |
|  |  | *Serratia liquefaciens* |  |  |  |  |  |  |
|  |  | *Pseudescherichia vulneris* |  |  |  |  |  |  |
|  |  | *Hafnia alvei* |  |  |  |  |  |  |
|  |  | Citrobacter braakii |  |  |  |  |  |  |

## **FIGURE S1**


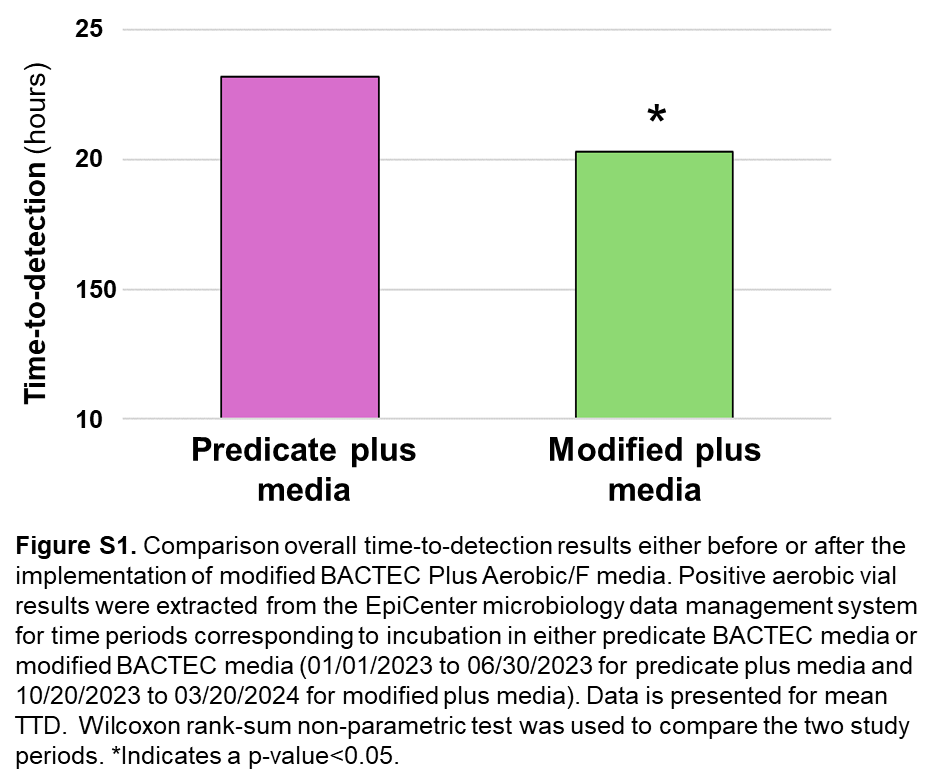


**FIGUR** SUPPLEMENTAL MATERIAL

## **FIGURE S2**


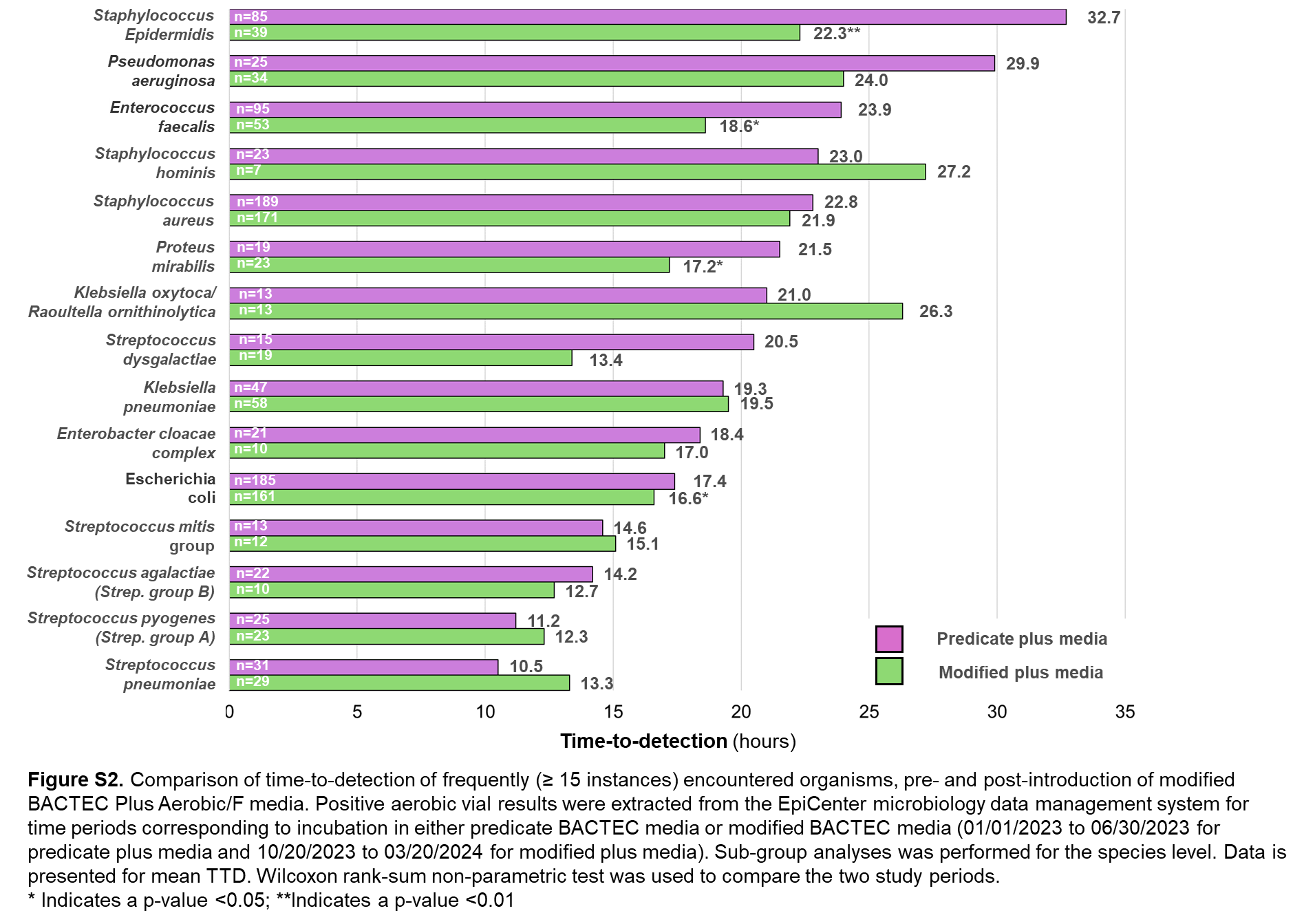

Supplement: Supplemental material — Tables S1 to S8; Fig. S1 and S2. [file spectrum.01969-25-s0001.docx]
